# The Role of Housing Environment and Dietary Protein Source on the Gut Microbiota of Chicken

**DOI:** 10.3390/ani9121085

**Published:** 2019-12-05

**Authors:** Shawna Marie Hubert, Morouj Al-Ajeeli, Christopher A. Bailey, Giridhar Athrey

**Affiliations:** 1 Department of Poultry Science, Texas A&M University, 2472 TAMU, College Station, TX 77845, USA; 2 Thorasic/ Head and Neck Oncology Research Department, The University of Texas M.D. Anderson Cancer Center, Houston, TX 77030, USA

**Keywords:** poultry, gut microbiota, free-range, caged, cage-free, diet, environment, bioinformatics, 16s rRNA

## Abstract

**Simple Summary:**

The gut microbiota—the community of microorganisms that colonize the gut—is now recognized as a key regulator of immune activity, metabolism, and welfare in all vertebrates, including poultry. The diet and environment can both influence the gut microbiota, but the extent of these changes is unclear in poultry, where diets and environments are important management tools. As the majority of U.S. egg production (>90%) has pledged to move to cage-free egg production by 2025, it is necessary to understand how much the diet and the rearing environment contribute to gut microbiota composition and function, and ultimately to health and production traits of chicken. We addressed this unknown by analyzing the gut microbiota community of laying hens with both the housing environment and diet as variables. We compared conventional cage systems against cage-free systems. In both environments, hens were fed a standard soy-based diet, versus an alternate soy-free diet. We found that cage-free environments generated higher gut microbiota diversity, and that the diet had a relatively lower effect on changing the gut microbiota. Our results highlight the difficulty of promoting consistent, beneficial gut microbiota across production systems or diet variations in commercial poultry conditions.

**Abstract:**

The gut microbiota of chicken has received much attention due to its importance for bird health, food safety, and performance. In the United States, the impending transition to cage-free housing environments has raised many questions about its consequences for poultry health, productivity, and welfare. Therefore, we investigated how housing environments and feed composition affect the poultry gut microbiome. Such data is necessary to inform the design of production systems that promote health and food safety. In this study, we investigated the cecal microbiome of both caged and cage-free laying hens that were fed either an industry-standard soy-based versus a soy-free diet. Caged hens were housed in standard industry-style layer cages with one bird per cage, and cage-free hens were housed in a poultry barn with an outdoor enclosed yard with multiple hens per pen. Our study showed significant differences in the gut microbiota between cage-free and caged environments. Cage free housing generated higher diversity compared to caged housing. Furthermore, we observed a synergistic interaction of soy-based feed in cage-free housing, as the cage-free soy group showed the highest alpha diversity, whereas the caged-soy group showed the lowest diversity overall.

## 1. Introduction

Poultry is a major source of wholesome (low fat, high protein) nutrition around the world, in addition to being one of the most economical of livestock production species [1]. Ensuring the health of laying hens and maintaining the economy of poultry products (eggs and meat) is crucial for global food security and sustainability. Two major upcoming shifts in U.S.-based poultry production models have emerged as significant challenges to be addressed within the next decade; firstly, the shift from caged to cage-free production systems prompted by consumer concerns about welfare, and secondly, the U.S. ban on antibiotic usage in livestock production [2]. These shifts have brought both the rearing environment and gut health into sharp focus. Even as poultry gut microbiota has been well investigated for several years [3,4,5,6], Kogut [7,8] has argued, for example, that we still lack information on how the microbiota interacts with the host immune system, especially in the context of nutrition or environment. In this study, we assessed the role of dietary protein source (conventional versus soy-free), as well as rearing environment (caged versus cage-free) in shaping the gut microbiota community of laying chicken.

The gut microbiota in vertebrates is recognized as an essential functional site for metabolic and immune health. The gut microbiota is an assemblage comprising hundreds of microbial taxa and has a vital role in the feed metabolism [9,10], stimulation of the immune system [11,12], and competitive exclusion of pathogenic organisms [13,14,15]. The digestive tract of chickens is short (total length of approximately 250 cm, 6–10× body length) compared to the digestive tracts of mammals (10–30 × body length), with a complete transit time of five to six hours. The shorter length of the chicken gastrointestinal tract allows for faster digestive processes and also microbiota composition that differ from other sections of the gastrointestinal track. The ceca, two blind sacs that serve as a site for fermentation and digestion, are considered crucial for avian health and immunity and are one of the best-studied foci for gut microbiota [16]. It is well known that various dietary factors, including feed composition, antibiotic growth promoters and dietary supplements [10,16,17] may alter the cecal microbiota composition. However, there is relatively limited knowledge about how such dietary factors interact with the rearing environment. For example, the bedding material or litter affects the gut microbiota composition [18,19,20]. Understanding this interaction is vital for determining the extent to which diets can be used to modulate gut microbiota, and whether microbiota differences elicited by the rearing environment supersedes or is superseded by dietary changes. Furthermore, it is also necessary to determine if such differences are qualitative, quantitative, or both, in terms of the presence and abundance of microbial taxa.

### 1.1. Role of Environment in Microbiota Structure

The commercial poultry rearing environment has seen several changes over the past century. While historical (pre-1960’s) production systems relied on cage-free barns with outdoor access, streamlined commercial production systems since the 1960s [21] have relied on battery cage production systems as the predominant model for egg production. However, changes in consumer awareness centered around animal welfare have induced major U.S. egg producers to shift to cage-free eggs by 2025. Cage-free and free-range housing typically offers more space per individual bird as well as access to the outdoors and exposure to natural lighting [22]. These changes have posed various challenges to the egg producers, ranging from biosecurity to skeletal health, or gut health. One of the critical questions that deserves deeper understanding is the role of rearing environment on gut microbiota. A recent study by Cui et al. [23], which characterized gut microbiota between caged and free-range hens, found differences in gut microbiota by age, but as the study depended on PCR-DGGE (Polymerase Chain Reaction - Denaturing Gradient Gel Electrophoresis) fingerprints, and not on sequence databases, it was not possible to assess taxon representation and abundance. Generally speaking, we would expect that the gut microbiota structure would differ between rearing environments, due to the differences in the litter or the soil with which the birds interact. For example, Torok et al. [20] showed that broilers raised on different bedding materials (sawdust, shavings, paper, etc.) displayed different cecal microbiota communities, which in turn was suspected to influence growth and health. Therefore, it is necessary to understand the extent of differences in the gut microbiota of birds raised on the same diet, but in different housing systems.

### 1.2. Role of Dietary Protein Source

In the poultry industry, the cost of feed is the most expensive component of production, accounting for 60–70% of costs. In the U.S., most poultry diets utilize the lowest cost ingredients, typically corn and soy as the sources of energy and protein, respectively, to keep the cost of nutrition low [24]. While soybean meal (SBM) represents over 40% of the poultry diet [25], concerns about the anti-nutritive properties of SBM [26,27], the high concentration of isoflavones on bird or human health [28], have necessitated the search for alternative feed sources that may reduce or replace SBM in poultry diets. Climate change and increased demands from fisheries production are also expected to increase costs [29,30]. In humans, it is well established that the source of dietary macronutrients (proteins, carbohydrates, lipids) significantly influences gut microbiota composition and function [31]. While our knowledge of these phenomena in chicken lags behind that of our understanding in humans, poultry studies on diets with varying sources of medium-chain fatty acids [32], or dietary fiber [33] show that diets modulate intestinal microbiota in chicken. However, no published studies have simultaneously assessed the roles and interaction of diets and rearing environment on gut microbiome in chicken. Finally, antibiotic growth promoters (AGP) also influence the gut microbiome [34], but with the ban on AGPs in the United States, it is necessary to characterize the extent to which diet and environment can be utilized as tools to modulate beneficial gut microbiota. Therefore, the motivation for the present study was to assess the differences in cecal microbiota arising from dietary protein source and housing environment.

In this study, we investigated the cecal microbiome of caged and cage-free Hy-line Brown laying hens reared on either a standard industrial soybean meal (SBM) based diet versus a diet that substituted cottonseed meal (CSM) for SBM. Our overall hypothesis was that the housing environment has a profound influence, compared to dietary protein source in altering the microbiota composition in chicken. Specifically, for the housing environment aspect, we expected that the cage-free environment would generate greater gut microbiota diversity irrespective of dietary protein source.

## 2. Materials and Methods

### 2.1. Study Population 

All laying hens in this experiment were a subset of hens used in the study reported by Al-Ajeeli et al. (IACUC 2017-0072) [25]. Briefly, the study started with 246 11-week old Hy-Line Brown pullets, divided into either the caged or cage-free group at 20 weeks of age (120 for caged, and 126 for cage-free). The hens in the cage-free treatments had continuous access to the outdoors, in a way that would be consistent with either cage-free or free-range definitions in the U.S. In the United States, free-range is defined as a housing system where hens are able to move both vertically and horizontally and have ready access to outdoor environments during the laying cycle. Caged and cage-free hens were housed in neighboring barns designed to be utilized for those specific housing types, as biosecurity requirements are unique for each. Within each of these housing environments, the hens were randomly assigned to either of the two dietary treatments—namely the standard SBM based feed, versus the CSM based feed. In the caged group, 20 hens (in individual cages, 1549 cm^2^ per hen) were assigned to a block, whereas in the cage-free group, hens were divided across pens, holding 21 hens per pen with 9509 cm^2^ per bird. Pine shavings were used as bedding material in the cage-free treatments. The diets were energetically equivalent and were formulated based on the Hy-Line Brown management guide (source: Hy-Line International). Additionally, the experimental feeds (mashed) fed to the caged or cage-free hens were from the same batch. Water supply was common to both housing styles. Hens in all treatment combinations were provided ad libitum feed and water. Details of diets and nutrient composition were previously published in in Al-Ajeeli et al. [25], and also reproduced here as Supplementary Table S1. Four main diet x environment treatment combinations were defined as Caged Soy (CS), Caged Soy-Free (CSF), Cage-Free Soy (CFS), and Cage-Free Soy-Free (CFSF).

At the end of the live animal study (hen age = 55 weeks), four to eight hens per treatment combination were randomly selected from across blocks/pens. For the caged environment group, we sampled eight hens per diet treatment, whereas, for the cage-free group, we sampled four hens per diet treatment. The disparity in the number of replicates between treatments was due to fewer hens available in the cage-free group at the end of the performance study. In total, 24 hens were sampled for microbiota analysis. As the sampled individuals came from different pens, each hen was considered as a unit of replication.

### 2.2. Sample Collection and Storage

A total of 24 Hy-line Brown laying hens from the four treatment groups were euthanized by exposure to CO_2_ gas, followed by cervical dislocation. Euthanasia procedures were performed using protocols approved by the Texas A&M Institutional Animal Care and Use Committee (IACUC #2017-0073). Following euthanasia, cecal sacs were collected within 30 min post mortem using sterile dissection tools. The cecal content and tissue were collected and stored separately in RNALater (ThermoFisher Scientific, Waltham, MA, USA) at a 1:5 ratio, following manufacturer protocols. These samples were stored at 4 °C for 24 h and then removed from the RNALater and stored at −80 °C, until DNA isolation.

### 2.3. 16S rRNA Gene Amplification and Sequencing

We isolated DNA from Cecal samples using the MoBio PowerFecal kit according to the manufacturer’s protocol (MO BIO Laboratories, Inc., Carlsbad, CA, USA). Cecal material was homogenized using a bead beater (BioSpec Products Mini-BeadBeater, Vernon Hills, IL, USA). High-quality DNA (20 ng) was used for PCR amplification of bacterial 16S rRNA gene sequences, using Q5 High-Fidelity DNA polymerase (NEBNextâ High-Fidelity 2X PCR Master Mix, New England BioLabs, Ipswich, MA, USA). We used a 15-cycle PCR first to amplify the 16s sequence (in triplicate) followed by 7-cycle PCR to add the Illumina barcodes. The V4 primer pair was explicitly chosen to avoid amplification of eukaryotic 18S rRNA gene sequences (Hyb515F_rRNA: 5’-TCGTCGGCAGCGTCAGATGTGTATAAGAGACAGGTGYCAGCMGCCGCGGTA -3.’, Hyb806R_rRNA: 3’-TAATCTWTGGGVHCATCAGGGACAGAGAATATGTGTAGAGGCTCGGGTGCTCTG-5’ [35]. Barcoded amplicons were cleaned up using Ampure beads (Beckman Coulter, Indianapolis, IN, USA). Library preparation and sequencing of samples was performed at the Genome Sequencing and Analysis Facility (GSAF, University of Texas, Austin, TX, USA). Amplicons were sequenced in 2 × 250 bp paired-end mode on an Illumina MiSeq platform (Illumina, San Diego, CA, USA).

### 2.4. Microbiota Analyses

We processed the raw sequence reads using the Mothur software, version 1.38 [36]. Briefly, we filtered the sequences by base quality and joined paired-end reads using the make.contigs command. Sequences of incorrect length and with ambiguous base calls were removed using the screen.seqs command. The remaining sequences were aligned against the SILVA database (release 123) [37,38] using the NAST (Nearest Alignment Space Termination) algorithm [39] and screened for homopolymers greater than eight bases. Chimeras were removed with UCHIME [40,41], and sequences were classified against the SILVA taxonomy [38] using the Bayesian classifier [42]. Sequences that classified to Eukaryota, Archaea, chloroplast, mitochondria, or unknown were excluded from further analyses. Sequences were clustered into operational taxonomic units (OTUs) of 97% sequence similarity using the average neighbor algorithm (default). Rarefaction curves for the observed number of OTUs were generated in Mothur using 1000 randomizations. Weighted and Unweighted Unifrac analyses were also performed using the Mothur software. α diversity and the impact of experimental variables on community differences were analyzed and compared using the Phyloseq (version 1.14.0) [43] and vegan (version 2.4-2) packages in the R software environment [44]. Principal coordinates analysis (PCoA) and plots were created in R. Permutational multivariate analysis of variance (PERMANOVA) with linear model fitting [45,46,47] using the “Adonis” function in the vegan package was performed to test how well the groupings, based on the metadata factors, accounted for the variation between the samples. Statistical tests of α and β diversity (PERMANOVA, metastats, LEfSe) were performed on pairwise datasets [48], comparing either the diet or environment or diet x environment combination as a variable. Additionally, as we used the SILVA taxonomy database for classification, we did not perform analyses of functional enrichment with PICRUSt [49], which requires that the taxa are classified using the Greengenes taxonomy database.

## 3. Results

Sequencing of 16s amplicons generated a total of 261,093 raw reads, of which 220,891 reads remained after quality filtering, and were used for analysis using the Mothur protocol. After completion of all quality control steps (duplicate removal, chimera removal, removal of non-prokaryotic sequences), 219,188 sequences remained, with an average 9132 sequences per library (sample) and were used for operational taxonomic unit (OTU) classification and statistical analyses.

**Figure 1:**
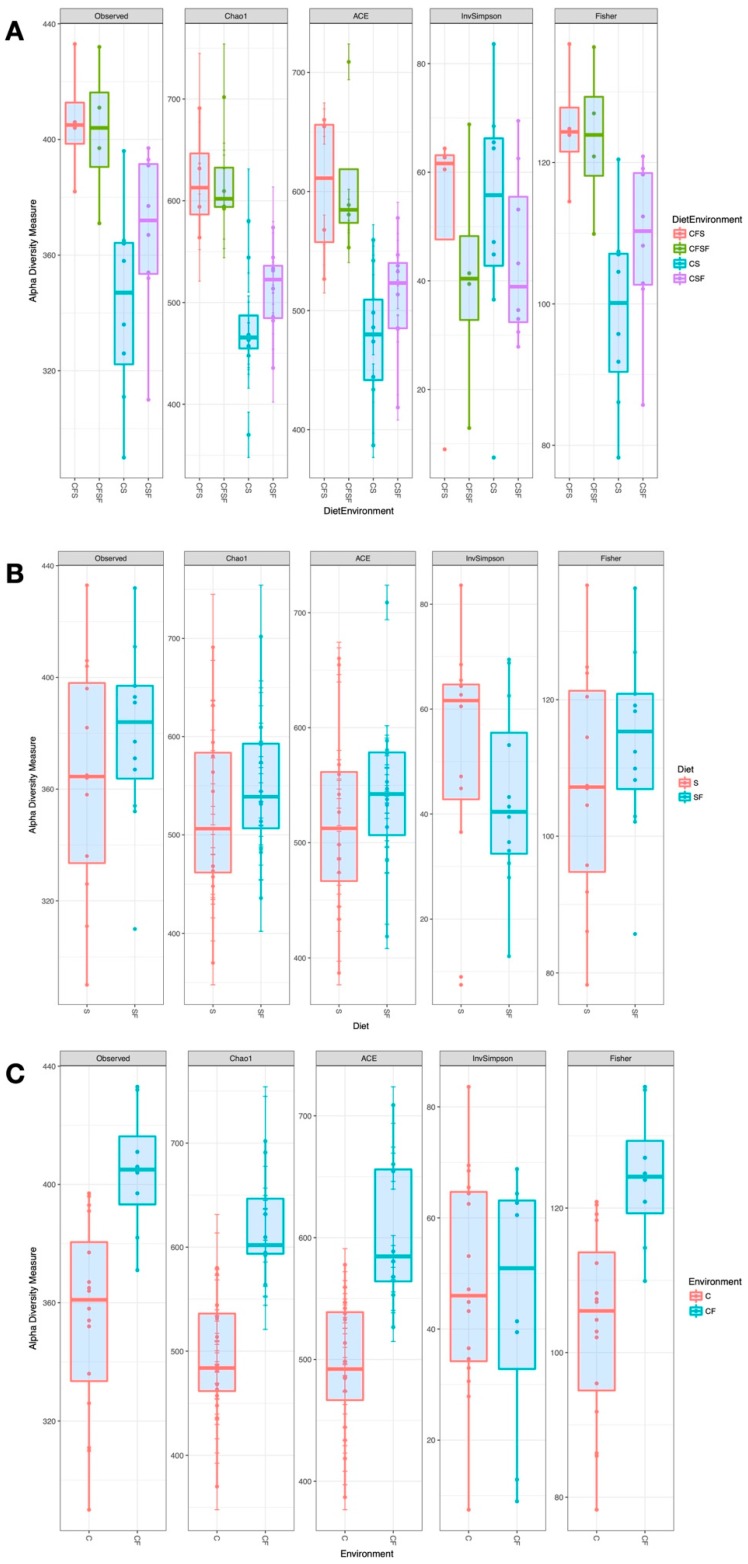
Box plots of Alpha Diversity measures from the cecal microbiota of laying hens. The plots show the comparison of estimates based on a grouping by (**A**) Diet x Environment—Caged soybean meal (CS), Caged cottonseed meal (CSF), Cage-Free soybean meal (CFS), Cage-Free cottonseed meal (CFSF), (**B**) Dietary protein source—soybean meal (S) versus cottonseed meal (SF), and (**C**) Environment—Caged (C) versus Cage-Free (CF).

A total of 1497 unique OTUs were observed across the treatments, which were classified up to the genus level. Individual samples showed some variation in the number of taxa identified, ranging from 290 to 432 among libraries. Estimates of alpha diversity showed that overall, the cage-free groups (CFS and CFSF) had higher microbiota richness compared against caged treatments, irrespective of protein source (Figure 1A). Dietary protein source was not a predictor of alpha diversity; although the median value was consistently higher for the soy-free group, the differences were not statistically significant (Figure 1B). The microbiota communities in hens raised in cage-free environments showed higher alpha diversity (Figure 1C), and except for Inverse Simpson index, the other indices (Chao1, ACE, Fisher) were statistically different between caged and cage-free environments (Kruskal-Wallis Chi-Square test, P < 0.005, df = 3). Pairwise comparisons showed that the alpha diversity estimates between CFS and CFSF treatments were not significantly different (Wilcoxon Sum Rank Test, P = 0.866).

Similarly, caged treatment groups (CS and CSF) were not different from each other (P > 0.2). All pairwise comparisons of cage-free groups against caged groups were statistically significant (Wilcoxon Sum Rank Test, P < 0.05, Benjamini-Hochberg adjusted for multiple comparisons). The CS treatment had the lowest alpha diversity of all experimental groups.

**Figure 2:**
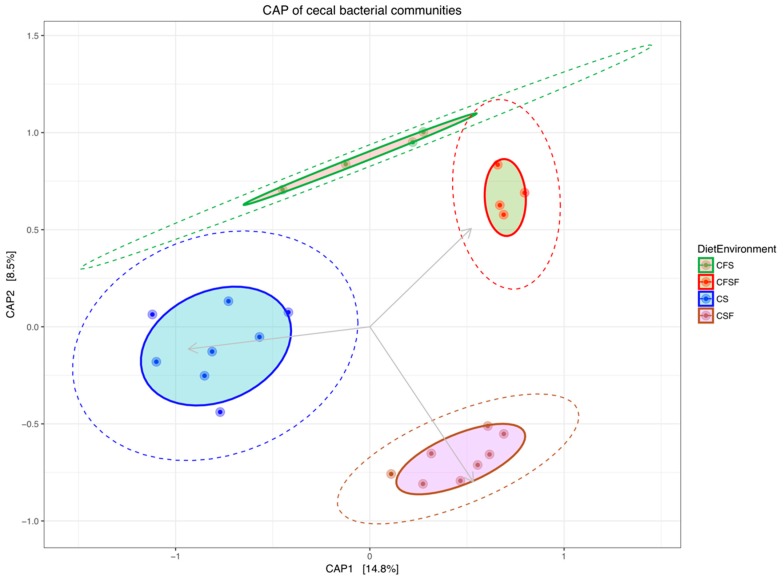
An ordination plot based on a constrained analysis of principal coordinates (CAP) showing the clustering of cecal samples by treatment grouping (Diet x Environment—Caged soybean meal (CS), Caged cottonseed meal (CSF), Cage-Free soybean meal (CFS), Cage-Free cottonseed meal (CFSF)). Each treatment group clustered distinctly from others. The cluster of samples are encircled by a 95% Euclidean distance ellipse (solid), and a 75% normal multivariate distribution (dashed).

β-diversity, calculated using the permutational analysis of variance (PERMANOVA) using the diet x environment as grouping factor (CS, CSF, CFS, CFSF), was significantly different among the groups (F = 2.53, P = 0.001, df = 3). The weighted and unweighted UNIFRAC (Unique Fraction) analyses also confirmed these communities to be significantly different. These differences in communities were also observed using the Constrained Analysis of Principal Coordinates (CAP) ordination analysis, based on the Bray-Curtis distances (Figure 2). Each diet x environment group clustered distinctly from each other. However, the ellipse encompassing the 95% normal multivariate distribution around the individual samples showed an overlap only between the CFS and CFSF groups.

**Figure 3:**
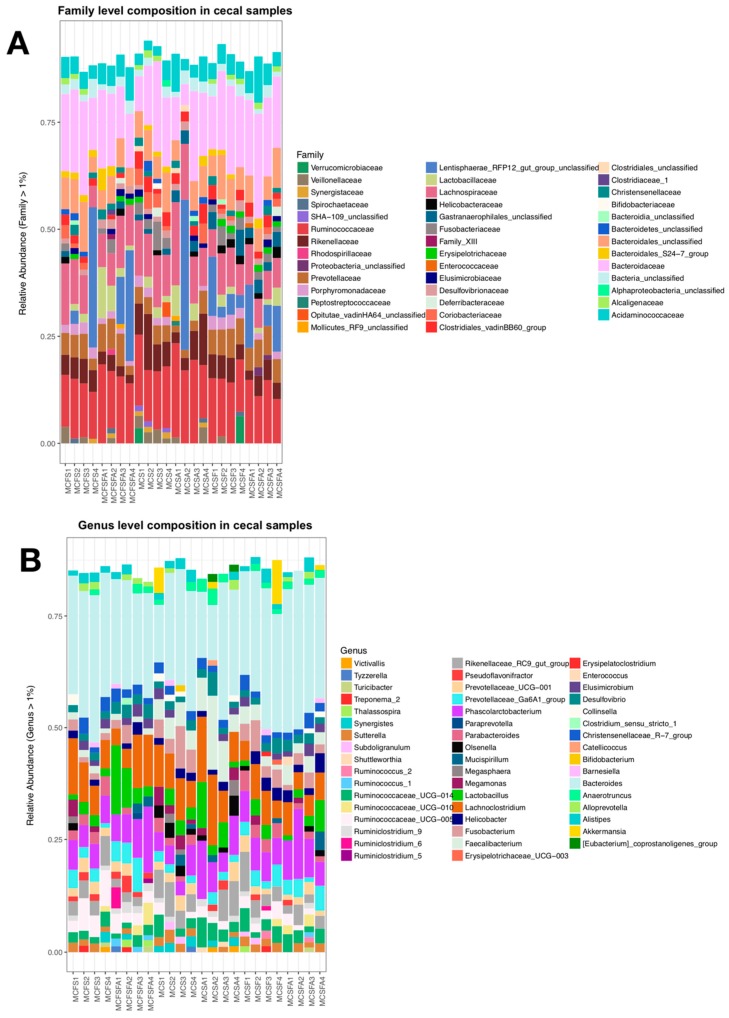
Column plots showing the distribution of taxa in the ceca of laying hens raised on different diets and housing environments. (**A**) shows column plots of family-level classifications by the sample (each column), and (**B**) shows genus-level classifications by the sample. Sample labels represent treatments with the following abbreviations Caged soybean meal (CS), Caged cottonseed meal (CSF), Cage-Free soybean meal (CFS), Cage-Free cottonseed meal (CFSF).

When comparing across groups, we found that 697 OTUs were shared among the four groups. While the CS and CFSF did not have any unique taxa, the other two groups showed 29 (CSF) and 80 (CFS) OTUs found exclusively in those groups. However, there was considerable intra-group variation in the OTU distribution among individual samples, both at the family and the genus levels. *Bacteroidaceae* and *Ruminococcaceae* were the most abundant families, whereas *Bacteroides* was the most abundant taxon, followed by *Lachnoclostridium* (Figure 3A,B).

**Figure 4:**
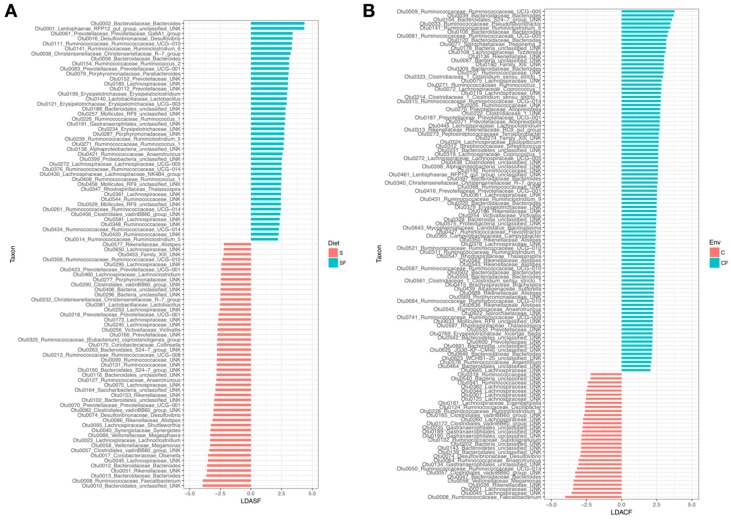
Plots showing the differential enrichment of taxa among treatments based on linear discriminant analysis. (**A**) shows the relative enrichment of taxa based on the diet treatments, and (**B**) shows the relative enrichment of taxa based on the housing environment.

Next, we compared differential enrichment of taxa (linear discriminant analysis) using only the diet or environment as differentiating factors. We focused on these comparisons given that the alpha diversity indices did not show significant separation between the CFS and CFSF groups, or between the CS and CSF groups. While soy versus soy-free microbiota communities were not significantly different, we were interested in determining specific taxa that are differentially enriched. The Linear Discriminant Analysis (LDA) analysis showed 93 differentially enriched taxa between the soy and soy-free treatments (LDA > 2, P < 0.05), whereas the caged versus cage-free comparison showed 120 differentially enriched taxa. In the soy versus soy-free comparisons, 49 taxa were enriched in the soy diets, of which *Faecalibacterium*, *Bacteroides* (OTU0013), and *Olsonella* were among the top enriched. In the soy-free treatment *Bacteroides* (OTU0002), *Desulfovibrio*, and *Ruminoclostridum* were among the top enriched (Figure 4A). In the caged versus cage-free comparison, the distribution of enriched taxa was more lopsided. The caged group showed 31 that were taxa significantly enriched (LDA > 2, P < 0.05), whereas the cage-free group had 91 taxa that were significantly enriched. In the caged group, *Faecalibacterium*, *Megamonas*, and *Bacteroides* (OTU0047) were among the top enriched, whereas in the cage-free group *Ruminococcacceae* UCG_005, *Bacteroides* (OTU0039), and *Pseudoflavonifractor* were the top enriched (Figure 4B).

**Figure 5:**
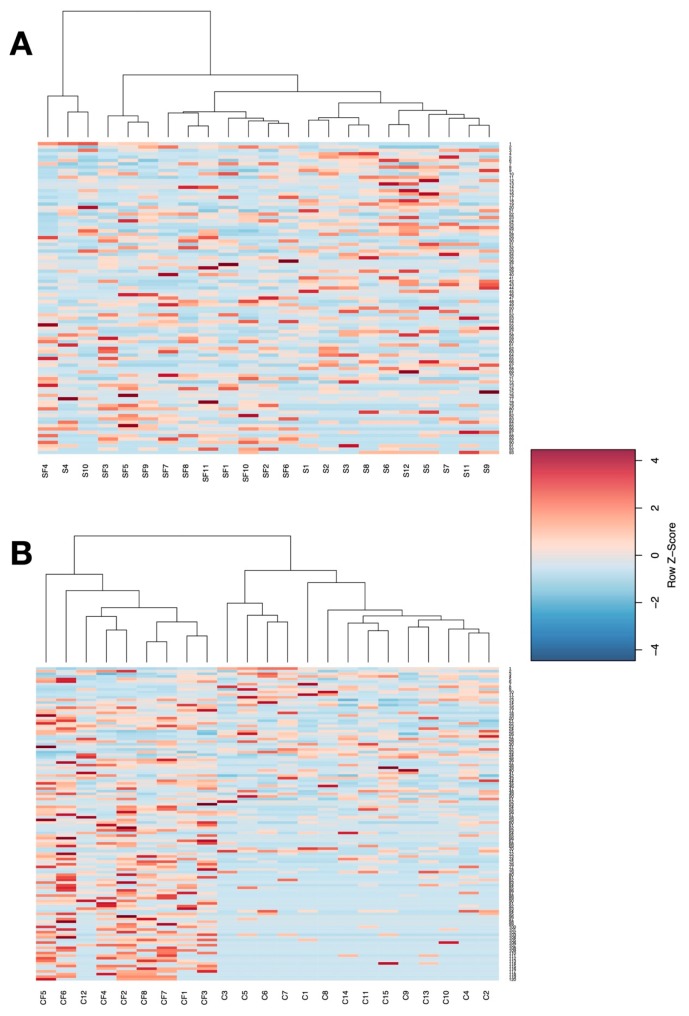
Heatmap showing the relative abundance of microbial taxa and grouping of samples based on hierarchical clustering analysis. Each column represents one sample. (**A**) shows clustering when analyzed by diet—soybean meal (S), cottonseed meal (SF), and (**B**) shows clustering when analyzed by the housing environment—Caged (C), Cage-Free (CF). Plots show a more obvious separation and clustering based on the housing environment.

Utilizing the differentially enriched taxa in hierarchical clustering analysis, we found that there was a high degree of intra-group variation in the soy-free versus soy comparison (Figure 5A). However, the cage-free group clustered together, and also showed a more consistent pattern of taxon enrichment (Figure 5B). This intra-group consistency was also observed in the caged treatments.

## 4. Discussion

In the previously published study [25], we showed that egg production did not differ significantly between diets, but significantly different performance metrics were observed between caged and cage-free environments. Additional details for performance study are reported in Al-Ajeeli et al. [25]. Following this, here we have investigated the interplay of diet x environment regarding the hen microbiome and possible significance for the poultry industry.

### 4.1. Lower Richness and Enrichment of Taxa in Caged Environments

The caged soy group had the overall lowest alpha diversity of all the treatments, and furthermore, 31 taxa were enriched in caged environments only. Among these enriched taxa, there were several taxa of potential interest to gut health. For example, *Megamonas* (Veillonellaceae) strains (type II) have been found to be positively correlated with *Campylobacter jejuni* in poultry [50]. In our study, we were unable to distinguish between *Megamonas* type I and type II sequences, but the association with *C. jejuni* exclusion in caged rearing environments is notable (R^2^ = 0.22). Coprophagy in caged housing may also be a contributor to the patterns of microbiota diversity observed in this diversity. Waldburg-Zeil et al. [51] showed that chickens prefer to avoid feeding on feces if they have a choice. The increased spatial footprint and diversity of substrate in cage-free or free-range environments may support the natural fecal-avoidance behavior and in turn contribute to the higher microbial diversity represented in their GI tract. However, we did not track coprophagy in either housing system and we are thus unable to determine how much of a factor coprophagy is between these environments.

### 4.2. Soy-Based and Cotton-Seed-Based Diets Generate Comparable Microbiota Diversity

Previous studies on the influence of cottonseed meal as an alternative to soybean meal on poultry gut microbiota have been limited, perhaps due to the gossypol toxicity associated with cottonseed meal [52,53,54]. Sun et al. [17] reported no effects on gut microbiota diversity but did find increased colonization of *Lactobacillus*, with the inclusion of fermented cottonseed meal. With the recent USDA deregulation [55] of a transgenic cotton variety with ultra-low gossypol levels [56] as a food source, the use of cottonseed meal in poultry diets may become a more common practice. Further studies are required to determine if the different bacterial communities we found to be enriched in CSM based diets have a role in metabolism and detoxification of gossypol.

In our study, we found that soy-free diets promoted higher microbiota diversity compared to soybean-based diets, and this difference was most pronounced in caged environments. In cage-free environments, the differences between the diets were less remarkable. When comparing taxa enriched among diet treatments (regardless of environment), we observed an almost equal number of taxa differentially enriched, which suggests that different microbial communities specialize in the utilization of the distinct protein sources.

### 4.3. Cage-Free Environments Support Higher Chicken Microbiota Diversity

In this study, we found that cage-free environments generated greater microbiota diversity, regardless of dietary protein source. Previous studies have reported that hens housed in conventional cages exhibited the lowest microbiota diversity, compared to aviary systems, but that the low diversity did not predict colonization of *Salmonella* [57]. We also found a greater proportion of taxa enriched in cage-free environments, compared to caged environments. Additionally, the greater microbiota diversity of the outdoor environment may mask the microbiota differences emerging from the diet alone. These results suggest that the outdoor access and contact with soil and natural vegetation are all potentially crucial in increasing gut microbiota diversity. If greater diversity translates into an improved immune and metabolic performance, (as reviewed in [58,59]), these data have implications for managing gut health in cage-free and organic production systems. Our results also suggest that dietary approaches to modulate gut microbiota may be challenging in outdoor environments. On the other hand, some researchers have argued, (eg reviewed in [60]), that the costs of increased microbiota diversity are at odds with animal production performance. For instance, the microbiota may compete with the host for nutrients. However, the increasing emphasis on gut health with the ban on antibiotic growth promoters [2,61] may reverse this mindset.

Our finding of significantly different gut microbiota communities between housing systems is not surprising given previous work on this topic. One notable aspect of this study was that the hens were raised in their environment for the entire duration of the study (44 weeks), which may be one reason for the stratification of microbiota differences between environments. Poultry housing environments are known to influence microbiota diversity and structure, as reviewed in Kers et al. [18]. For example, organic production models with outdoor access were found to enrich *Clostridium perfringens* and *Lactobacillus* [62]. Xu et al. [63] reported a lower proportion of *Bacteroidetes* in caged Dagu chicken (compared to free-range), but our study did not find notable differences in *Bacteroidetes* between environments or diet. However, several earlier studies [62] did not utilize 16s amplicon sequencing to characterize diversity, which makes it difficult to compare our results across studies. Furthermore, the breed and host genotype have can both influence the gut microbiota communities [64,65,66]. Therefore, the extent to which the poultry breed (Hy-line Brown, Broilers, heritage varieties) influences the gut microbiota community across environments limits our ability to draw common themes from studies using different breeds and production systems.

Finally, cage-free and free-range environments experience different microbiota dynamics due to their exposure to other environmental factors, including natural lighting and rainfall. Light intensity and natural photoperiods may reinforce unique microbiota communities, some of which may also oscillate in synchrony with the host biological rhythms [67,68]. These phenomena have been well documented in mouse studies, and we recently demonstrated that photoperiods influence both microbiota diversity and structure in Hy-line Brown layers [69]. Taken together, the synergistic contribution of cage-free environments is likely to have a more significant contribution to poultry gut microbiota and health than the impact of the soil and litter differences when compared to caged environments.

## 5. Conclusions

Our study showed significantly different cecal microbiota profiles in laying hens raised in caged- versus cage-free housing systems. Limited differences were found based on the source of protein in their diet, but soybean-based diets generated profoundly different microbiota profiles between housing environments. The higher alpha diversity in cage-free environments may be a result of greater substrate diversity and reduced coprophagy. Whether these differences in diversity translate into differences in immune maturation and gut health needs to be further investigated. Finally, due to the large effect of the environment in generating gut microbiota profiles, our results highlight the difficulty of promoting or engineering beneficial poultry gut microbiota across production environments, or diet variations. On the other hand, the relative consistency of gut microbiota in the same environments, regardless of dietary protein source, provides an avenue for the application of production-system specific approaches for microbiota management.

## Supplementary Materials

The following are available online at https://www.mdpi.com/2076-2615/9/12/1085/s1.
