# Supplementary material for: The Role of Housing Environment and Dietary Protein Source on the Gut Microbiota of Chicken
Source: Animals (Basel). 2019 Dec 5;9(12):1085. doi: 10.3390/ani9121085 (PMC6940977; doi:10.3390/ani9121085)
Supplement: Supplementary file 1 [file animals-09-01085-s001.pdf]

Supplementary Table 1: Table of the nutritional compositions of the experimental diets used in this study. The table is extracted from the published paper by Ajeeli et al, 2017 (Poultry Science).

Table 1. Composition and nutrient levels of corn/soybean and corn/soybean-free diets from 19 to 55 wk of age.

Ingredients	Soybean% 19 to 38 wk	Soybean free% 19 to 38 wk	Soybean% 39 to 44 wk	Soybean free% 39 to 44 wk
Corn	64.11	46.99	65.25	42.85
Dehulled soybean meal	20.04	-	21.07	-
Cottonseed meal	-	14.94	-	15.00
Corn gluten meal	0.38	1.38	0.31	1.63
Corn dried distiller grains with solubles	-	15.00	-	15
Wheat midds	-	3.59	-	8.65
DL-Methionine 98%	0.35	0.36	0.17	0.18
L-Threonine 98%	-	0.03	-	0.05
Lysine HCL	0.24	0.66	-	0.42
Fat—animal-vegetable blend[5]	2.75	4.73	1.76	4.75
Limestone	9.76	9.92	9.45	9.62
Mono-Dical PO4	1.66	1.65	1.32	1.27
Salt	0.29	-	0.38	0.06
Sodium bicarbonate	0.12	0.44	-	0.23
Trace minerals[1]	0.05	0.05	0.05	0.05
Vitamins[2]	0.25	0.25	0.25	0.25
**Calculated nutrient composition (%)**				
ME(kcal/kg)	2911	2911	2867	2867
Crude protein	16.5 (15.5)[4]	16.5 (16.2)[4]	16.75(14.96)[4]	16.75(17.16)[4]
Crude fat	4.58	7.36	3.62	7.43
Crude fiber	1.71 (2.6)[4]	4.18 (5.7)[4]	1.76(3.06)[4]	4.51(8.56)[4]
Calcium	4.08	4.08	3.91	3.91
Phosphorous	0.68	0.78	0.62	0.73
Available phosphate	0.45	0.45	0.38	0.38
Digestible methionine[3]	0.58	0.58	0.41	0.40
Digestible lysine[3]	0.90 (1.0)[4]	0.90 (0.99)[4]	0.74(0.79)[4]	0.73(0.90)[4]
Digestible TSAA[3]	0.80	0.80	0.64	0.63
Xanthophyll (mg/kg)	12	12	12	12
Electrolytes (meq/kg)	170	170	170	170
Sodium	0.17	0.20	0.20	0.20
Chloride	0.26	0.18	0.17	0.17

[1]Trace minerals premix added at this rate yields (mg/kg): zinc, 60.0; manganese, 60.0; iron, 60.0; copper, 7.0; iodine, 0.4.

[2]Vitamin premix added at this rate yields (per kg): vitamin A, 11 IU; vitamin D_3, 3850 IU; vitamin E, 45.8 IU; menadione, 1.5 mg; B_{12}, 0.017 mg; biotin, 0.55 mg; thiamine, 2.93 mg; riboflavin, 5.96 mg; d-pantothenic acid, 20.17 mg; B_6, 7.15 mg; niacin,45.8 mg; folic acid, 1.74 mg; choline, 130.3 mg.

Standardized digestibility coefficients for cottonseed methionine, lysine, and TSAA were 0.73, 0.67, and 0.73.

[4]Nutrient analysis was performed by Experiment Station Chemical Laboratory, University of Missouri.

[5]Griffin Industries, Bastrop TX.
